# Does Emotional Working Memory Training Ameliorate Anxiety and Depression? A Meta-Analytic Review

**DOI:** 10.3390/brainsci16010030

**Published:** 2025-12-25

**Authors:** Jiehang Ou, Hong Mou, Ting Zhou, Yingying Wang

**Affiliations:** 1 School of Psychology, Shanghai University of Sport, No. 650 Qingyuanhuan Road, Yangpu District, Shanghai 200438, China; 2 Center for Exercise and Brain Science, Shanghai University of Sport, No. 650 Qingyuanhuan Road, Yangpu District, Shanghai 200438, China

**Keywords:** emotional working memory, anxiety, depression, meta-analysis, cognitive training, N-back training

## Abstract

Depression and anxiety disorders are associated with deficits in several cognitive domains. This meta-analytic review assessed the effects of emotional working memory training (eWMT) on depression and anxiety and their related emotional and cognitive domains. Eligible studies were assessed for changes in depression, anxiety, emotion and cognition after eWMT. Methodological quality was assessed using Cochrane Collaboration’s guidelines, and random-effects models aggregated the results of individual studies. Of 1314 studies identified, 16 were included (883 participants; mean age range, 14.35–68.79 years; 70.44% female). Nine studies were high quality, seven were moderate quality, and none were low quality. There was relatively high heterogeneity across studies and study populations. The eWMT significantly reduced depression (standardized mean difference [SMD], −2.04; 95% confidence interval [CI], −3.68 to −0.39; *p* = 0.02) and anxiety (SMD, −0.44; 95% CI = −0.23 to −0.17; *p* < 0.001) and significantly enhanced reappraisal (SMD, 0.38; 95% CI, 0.11 to 0.66; *p* = 0.03) and working memory capacity (SMD, 0.31; 95% CI, 0.10 to 0.53; *p* < 0.001), with no significant effect on rumination. Training frequency, training environment, and type of control group differentially affected working memory capacity. Our results demonstrated that eWMT alleviated depression and anxiety, but not rumination, and improved the related factors of reappraisal and working memory. Given the limited number of studies and substantial heterogeneity in the data, further research is needed to support these results.

## 1. Introduction

Mental illness represents a significant global health burden, affecting both individuals and society at large [1]. Among mental health conditions, depression is the leading contributor to the disease burden [2], while anxiety is the most prevalent mental health disorder [3]. The overall prevalence of depression and anxiety among university students is as high as 33.6% globally [4], highlighting the widespread nature of these conditions. A critical insight is that current treatments inadequately address key underlying vulnerability factors of mental illness, resulting in incomplete remission or, when remission occurs, continued risk for new episodes [5]. Although cognitive impairments in concentration, memory, and attention were once regarded as secondary to affective symptoms, results of recent neurobiological and cognitive research suggest that reduced cognitive control over information in working memory may represent a central psychological vulnerability factor [6,7,8]. Working memory (WM) is a finite-capacity system that reflects the temporary activation of both attentional focus and the contents of conscious representations. Negative emotions are frequently linked to the activation of emotionally congruent representations within working memory [9]. Consequently, cognitive control—particularly the regulation of working memory contents—may play a pivotal role in the recovery from negative emotional states. In this context, theories of vulnerability to depression and anxiety have long emphasized the importance of both cognitive processes and emotion regulation [10,11,12].

Cognitive control encompasses the processes that enable individuals to regulate, coordinate, and sequence their thoughts and actions in alignment with internally set behavioral goals [13]. Components of cognitive control include updating, shifting, and inhibition [14]. Many forms of effective emotional regulation require high levels of cognitive control. For patients with depression and anxiety, flexible and effective updating functions—such as monitoring and manipulating the contents of working memory (e.g., adding or removing information)—can help avoid rigid thinking, like rumination, by allowing them to delete negative or no longer goal-relevant content from working memory [14].

The regulation of emotions plays a crucial role in influencing the levels of depression and anxiety, with numerous studies highlighting deficits in emotion regulation among individuals with these disorders [15,16]. For instance, individuals with depression often employ emotion regulation strategies, such as rumination and suppression, which have been found to be less effective [15]. Rumination involves passively and repetitively focusing on distress symptoms and their possible causes and consequences [16]. Similarly, individuals with anxiety disorders often struggle with suppression and reappraisal. Reappraisal is a cognitive-linguistic strategy aimed at modifying emotional responses by altering thoughts and beliefs about a stimulus or situation [17,18]. Some researchers propose that emotion regulation represents a form of cognitive control [19], and a decline in this cognitive function may contribute to increased emotional difficulties in individuals with depression and anxiety, particularly through rumination and insufficient reappraisal when addressing negative content.

Recent research has confirmed that emotional working memory training (eWMT), as a form of computerized cognitive training, effectively enhances individuals’ ability to suppress task-related distractors and improves working memory [20,21,22]. Working memory is defined as the cognitive system responsible for temporarily holding and manipulating information necessary for ongoing tasks [23]. eWMT tasks are typically performed individually and offer significant advantages, such as low cost, high availability, and ease of use at home [24]. Furthermore, these tasks facilitate the storage of behavioral and neural training data on devices, allowing for user data tracking and real-time adjustments to the training process [25].

Unlike traditional working memory training (WM training), which typically aims to enhance working memory capacity using non-emotional stimuli such as digits or symbols [26], eWMT incorporating emotional stimuli—such as emotional vocabulary and facial expressions—may provide greater benefits for emotion regulation [27,28,29]. Specifically, eWMT trains participants to disregard negative emotional stimuli during memory tasks, thereby promoting the ability to disengage from or reframe emotional contexts in real-world situations. This training enhances emotion regulation skills and ultimately contributes to the reduction in anxiety and depression. For instance, it enables individuals to distance themselves from negative thoughts and their associated emotional responses or to redirect their attention, rather than becoming absorbed in negative emotions.

The referential theoretical framework of emotion regulation [30] can elucidate the potential pathways through which eWMT influences emotional control. It posits two primary categories of emotion regulation: model-based regulation and model-free regulation. Model-based emotion regulation refers to explicit emotion regulation strategies, such as reappraisal. Research has shown that eWMT can enhance cognitive flexibility and the capacity to manage emotional content. For instance, improved working memory can facilitate the process of updating and replacing negative emotions with positive ones [31]. Consequently, numerous studies have demonstrated that eWMT can improve emotion regulation by enhancing cognitive reappraisal [26,28]. In contrast, model-free emotion regulation is characterized by implicit control, involving unconscious assessment of emotional value. eWMT that targets implicit control focuses on reducing the impact of negative emotional material, thereby influencing emotional processing at a more automatic level.

While evidence supports the efficacy of eWMT in reducing symptoms of depression and anxiety [28,32], findings remain mixed [33,34,35], and meta-analyses on traditional working memory training suggest limited far-transfer effects [36,37,38]. This inconsistency highlights a key theoretical gap: the lack of clarity regarding eWMT’s specific mechanisms of action. Grounded in the dual-pathway model of emotion regulation [30], this meta-analysis proposes that eWMT may enhance model-based regulatory resources while dampening model-free, maladaptive processes. To directly test this model, the present review examines a predefined cascade of theory-derived outcomes: the enhancement of its putative cognitive target (working memory capacity), changes in specific emotion regulation processes (cognitive reappraisal and rumination), and the alleviation of core clinical symptoms (depression and anxiety). Although eWMT may indeed influence broader domains such as general wellbeing or other aspects of cognitive control (e.g., inhibitory control; [39]), these outcomes fall outside the scope of the current theoretical framework and are often reported in fewer studies, precluding robust quantitative synthesis at present. Therefore, by systematically synthesizing evidence across this focused theoretical cascade, the present review aims to test the integrative dual-pathway model, clarify the specific efficacy of eWMT within its proposed mechanisms, and provide a consolidated evidence base to guide future research and clinical application.

## 2. Methods

### 2.1. Protocol Registration

This review protocol was registered in the PROSPERO repository (registration number: CRD42024563632). This study followed the Preferred Reporting Items for Systematic Reviews and Meta-Analyses guideline. We have updated the title and data search timeframe in our registration information. The original title, “Is Emotional Working Memory Training Effective? A Meta-Analytic Review,” did not adequately reflect the objectives of our study. The revised title more clearly articulates our focus on investigating the potential role of eWMT in alleviating symptoms of depression and anxiety. The adjustment in the data search period is essential to include recent studies published since the original registration. This extension ensures that the analysis incorporates the latest evidence, providing a more up-to-date and relevant overview of the field. The completed PRISMA checklist is provided in the Supplementary Information.

### 2.2. Literature Search

We searched the Web of Science, PsycINFO, PubMed, and ScienceDirect databases for relevant research reports (articles and dissertations) from the earliest dates available to April 28, 2025. Our search strategy was informed by a previous meta-analysis on a related topic [36]. Key terms included: (1) working memory training; (2) n-back training; (3) dual tasking training; (4) memory updating training; (5) switching training; and (6) shifting training; in combination with the terms (7) emotional; or (8) affective (see the Table S1 for details). The search was conducted in April 2025. The search resulted in 855 articles identified in Web of Science, 353 in PsycINFO, 64 in PubMed, and 28 in Science Direct. We also checked the references in each of the collected articles and identified 16 additional articles.

### 2.3. Criteria for Inclusion and Exclusion

**Figure 1:**
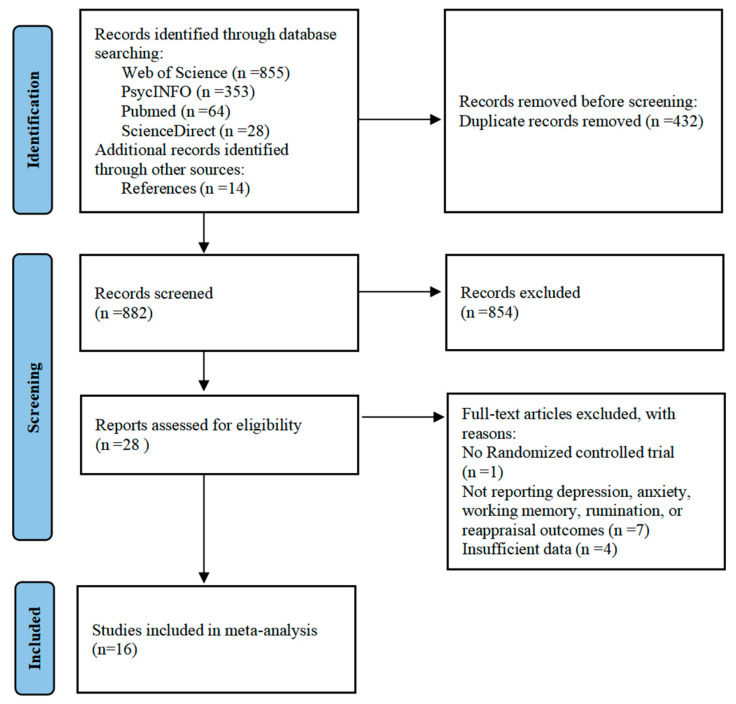
PRISMA flowchart.

We screened all articles identified from the databases. For a study to be included at this stage, the following criteria had to be met: (1) the study was a controlled trial with at least one training group and at least one control group and a pretest-posttest design; (2) the study includes interventions that only involve eWMT, while studies with multi-component training conditions (such as training that includes other types of tasks or a combination of medication and psychotherapy interventions) were excluded; (3) the results before and after training were measured (including at least one of depression, anxiety, working memory, rumination, or reappraisal), and the data were reported in a format suitable for meta-analysis; and (5) the study was published in the English language in a peer-reviewed journal. After the articles identified in the search were screened, 16 reports were included in this meta-analysis. The inclusion and exclusion process is summarized in Figure 1.

### 2.4. Data Collection

Data extraction was independently conducted by two reviewers (JH.O. and T.Z.) using standardized forms according to the exclusion and inclusion criteria. The reviewers screened the abstracts and then retrieved the required information, including the name of the first author, publication year, country, study design, participant characteristics (e.g., sex proportion, age, sample size, diagnostic situation), methods (e.g., randomization, blinding, measurements of outcomes, and tasks performed by control group participants), interventions (number of sessions, session time, training frequency, and training environment) and outcomes. For missing information, the authors of the respective articles were queried via email. The reviewers met every week to reach an agreement about the inclusion or exclusion of articles. Any discrepancies were brought to a senior investigator (YY.W.) and resolved by discussion.

### 2.5. Study Quality Evaluation

Study quality evaluation was independently conducted by two reviewers (JH.O. and T.Z.) according to the recommendations of the Cochrane Collaboration [40]. The risk of bias was assessed from seven aspects: random sequence generation (selection bias), allocation concealment (selection bias), blinding of participants and personnel (performance bias), blinding of outcome assessor (detection bias), incomplete outcome data (attrition bias), selective reporting (reporting bias), and other bias. The reviewers explicitly judged each of these seven criteria as exemplifying a low, high, or unclear risk of bias. Any discrepancies were brought to and resolved by a senior investigator (YY.W.). Articles of low quality were excluded from the meta-analysis.

### 2.6. Statistical Analysis

Data were pooled from randomized controlled trials using the mean difference in score changes (from baseline to post-intervention) between the eWMT and control groups for each outcome variable. Most of the studies reported means and standard deviations (SDs), permitting effect size estimation; otherwise, data were derived from the summary statistics reported in the articles, such as *t* or *p* values. The data from individual studies were then pooled in meta-analyses to estimate the overall effect of the training intervention. Meta-analyses were conducted to pool effect sizes where at least three studies provided data for an outcome. Summary effect estimates were calculated along with their 95% confidence intervals (CIs) and respective values for null hypothesis tests (e.g., eWMT training had no effect on rumination). The choice of statistical model was guided by the assessment of between-study heterogeneity. We quantified heterogeneity using the *I*^2^ statistic and the Cochran’s *Q* test. The *I*^2^ statistic describes the percentage of total variation across studies that is due to heterogeneity rather than chance. An *I*^2^ value of 0% indicates no observed heterogeneity, 25% low, 50% moderate, and 75% high heterogeneity. A *p*-value of less than 0.05 for the Cochran’s *Q* test was considered indicative of statistically significant heterogeneity. Based on this assessment: (1) A fixed-effect model will be applied if heterogeneity is negligible (*I*^2^ ≤ 50% and *Q* test *p* ≥ 0.10). (2) A random-effects model will be applied in the presence of substantial heterogeneity (*I*^2^ > 50% or *Q* test *p* < 0.05). Quantitative syntheses and meta-analyses were produced using Review Manager 5.4 software (RevMan 5.4). In cases of substantial heterogeneity (*I*^2^ > 50%), we planned to explore potential sources through pre-specified subgroup analyses.

To synthesize studies that used different measurement tools for the same construct, we employed standardized mean difference (SMD) as the effect size within a random-effects model. The specific procedure was as follows: outcomes were first coded by specific measure (e.g., “DASS-21,” “Complex Span Task”). Although subgroup analysis by measure was planned, most categories contained fewer than three studies using the identical tool, making such analysis uninformative. We therefore applied two practical strategies: (1) Prioritization of Predominant Measures: If >50% of studies for a construct used the same tool (e.g., DASS-21 for depression), data from that tool were prioritized to enhance comparability. (2) Inclusive Synthesis with Robustness Checks: Where no single tool predominated (e.g., WMC assessed by various span/n-back tasks), all validated measures were synthesized using a random-effects model, thereby incorporating measurement-related heterogeneity into the estimate. The robustness of results from both approaches was verified via sensitivity analyses (e.g., leave-one-out analysis, or analysis restricted to the prioritized measure).

Subgroup analyses were conducted to investigate the potential moderators influencing the primary outcomes, including number of sessions, training time, training frequency, type of control group, training environment, participant age (e.g., adolescents vs. older adults), stimulus valence in training (positive vs. negative/mixed), and stimulus type (e.g., words vs. facial images), and subject type, to provide information and suggestions for the design of future eWMT intervention research. Due to the limited duration of the training, the included studies were divided into two categories: short-term (≤10 times) and long-term (>10 times). Similarly, the training time was divided into two groups: small training dose (≤40 min) and large training dose (>40 min). The training frequency was also divided into two groups: lowtraining frequency (≤4 times/week) and high training frequency (>4 times/week). Finally, studies were categorized into two groups based on subject type: clinical population and general population. To ensure the analysis maintains adequate statistical power, subgroup analyses were conducted exclusively for groups comprising at least three studies, thereby minimizing the potential for bias arising from small sample sizes [41].

To test the robustness of the primary findings, we planned several sensitivity analyses. These included: (1) re-running the meta-analysis after excluding studies with a high risk of bias; (2) examining the influence of any outlier studies identified through visual inspection of the forest plot and statistical measures; and (3) using a fixed-effect model for comparisons originally based on a random-effects model.

### 2.7. Transparency and Openness

We followed the Preferred Reporting Items for Systematic review and Meta-Analysis framework [40]. All analyses were conducted using Review Manager 5.4 software (RevMan 5.4). The meta-analytic data set and relevant code for the present study are available from the corresponding authors upon request. The analyses in the present report were preregistered on International Prospective Register of Systematic Reviews (PROSPERO ID: CRD42024563632). We report how we determined our sample size (i.e., dependent upon available data from literature search), as well as all data exclusions, manipulations, and measures in the study (i.e., inclusion/exclusion criteria below).

## 3. Results

### 3.1. Characteristics of Included Studies

A total of 1314 records were identified through the search. Of these, 16 studies met the inclusion criteria and were included in the meta-analysis. The sample sizes of these studies ranged from 20 to 168 and included 883 participants in total. The mean age of participants ranged from 14.35 to 68.79 years. Participants were predominantly female (overall 70.44%, range 25.00–100%). Treatment duration ranged from 1 to 21 sessions. The characteristics of each study are shown in Table 1.

### 3.2. Study Quality

**Figure 2:**
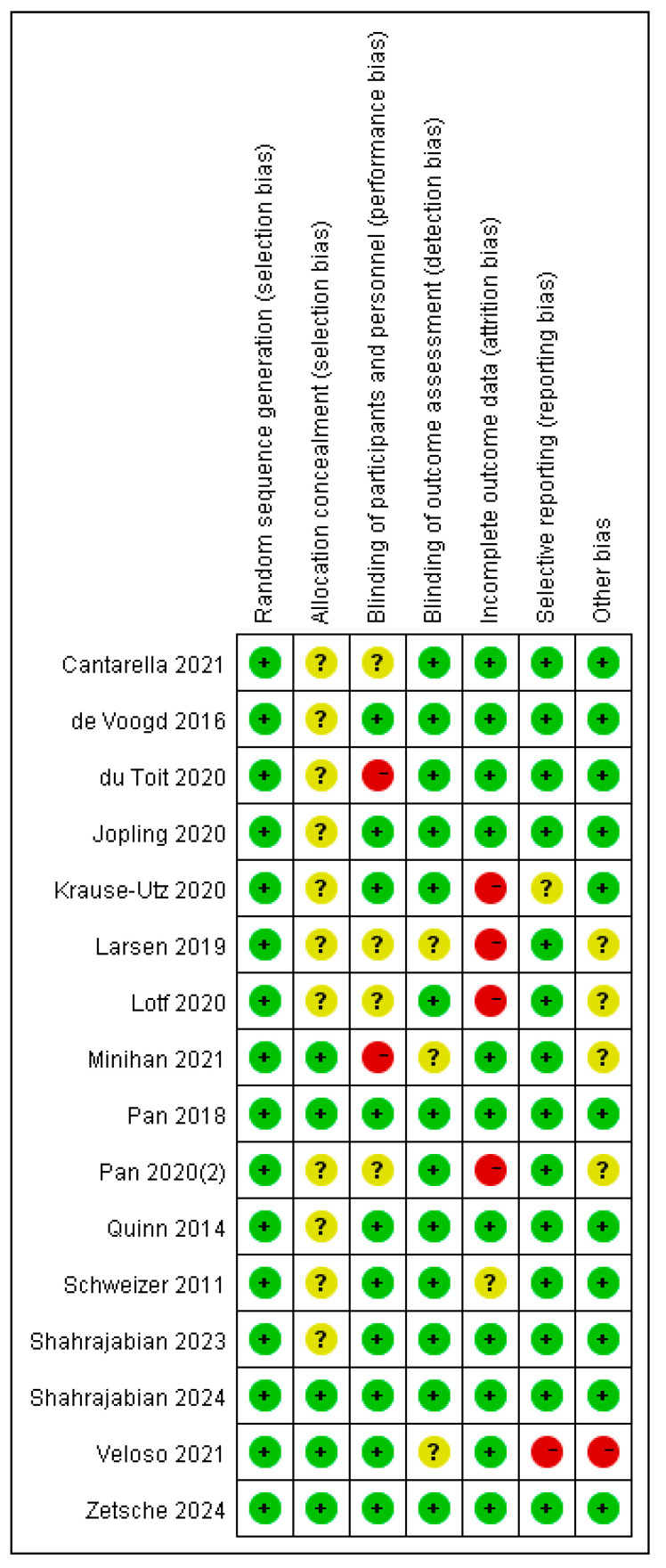
Summary of the risks of the examined biases for each included study. Green +, indicates low risk of bias; red - indicates high risk of bias, and yellow “?” indicates unclear risk of bias [20,24,28,32,42,43,44,45,46,47,48,49,50,51,52,53].

Based on Cochrane criteria guidelines, nine studies (50%) had high quality and seven studies (50%) had moderate quality. As no article of low quality was identified, all 16 studies were included in the quantitative analysis. Funding bias and selection bias were the most common risks of bias (Figure 2).

### 3.3. Outcomes

#### 3.3.1. Working Memory Capacity

**Figure 3:**
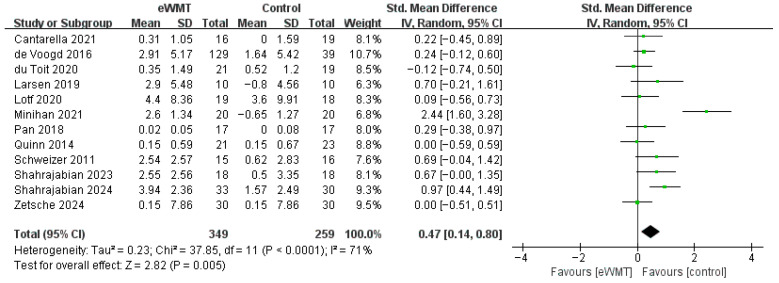
Forest plot of the effect of emotional working memory training on the capacity of working memory. Each square represents a study’s point estimate (size proportional to weight); the horizontal line shows the 95% confidence interval. Abbreviations: eWMT, emotional working memory training; IV, inverse variance; SD, standard deviation; CI, confidence interval [20,24,32,42,43,46,47,48,49,50,51,53].

The meta-analysis of twelve studies (n = 843) demonstrated that eWMT led to a significant, moderate improvement in working memory capacity compared to control conditions (SMD = 0.47, 95% CI = 0.14 to 0.79, *p* = 0.005). The CIs for the effect sizes of each study are given in the forest plot of Figure 3.

We observed substantial heterogeneity among the included studies (*I*^2^ = 71%), indicating considerable variation in the true effect sizes. Visual inspection of the forest plot identified the study by Minihan [32] as an outlier with an exceptionally large effect size (SMD = 2.44). A sensitivity analysis excluding this study yielded a smaller but still significant pooled effect (SMD = 0.31, 95% CI = 0.10 to 0.53, *p* < 0.001) with substantially reduced heterogeneity (*I*^2^ = 28%). This more conservative estimate is considered a more reliable representation of the effect of eWMT on working memory.

Subgroup analyses were conducted to explore sources of heterogeneity, with a focus on training parameters and participant characteristics. A notable finding was the substantial influence of a single study [32] on several subgroups; sensitivity analyses excluding this outlier are presented alongside the primary results (Table 2).

The relationship between training intensity and outcomes was complex. For the number of sessions, long-term training demonstrated a large but highly variable effect in the primary analysis. However, sensitivity analysis revealed a substantially reduced yet more precise and consistent effect (SMD = 0.80, 95% CI = 0.47 to 1.13, *p* < 0.00001; *I*^2^ = 0%). Conversely, short-term training was associated with a negligible and non-significant effect. A similar pattern was observed for total training time, where the large but non-significant effect of a large training dose became smaller and statistically marginal after excluding the outlier.

In contrast to intensity, training frequency was a more stable moderator. Both high- and low-frequency regimens yielded similar, significantly small-to-moderate effects. The effect of high-frequency training remained significant in sensitivity analysis (SMD = 0.32, 95% CI = 0.08 to 0.57, *p* = 0.01), albeit with a slight increase in heterogeneity.

Furthermore, sensitivity analyses confirmed the robustness of effects for one key subgroup: health populations. After excluding the influential study, subgroups showed significant effects with low heterogeneity (SMD = 0.28, *I*^2^ = 0%). Substantial heterogeneity persisted across clinical population subgroups, indicating that other unmeasured factors, likely related to participant characteristics, influence the intervention’s efficacy.

Finally, we note that the generalizability of our subgroup findings is constrained by the characteristics of the available literature. Meaningful comparisons for several theoretically important moderators, training environment (online vs. laboratory), control type (active vs. passive), participant age groups (e.g., adolescents vs. older adults), stimulus valence in training (positive vs. negative/mixed), and stimulus type (e.g., words vs. facial images)—were not feasible. This was due to an insufficient number of studies (k < 3) in one or more subgroups for each comparison. The available studies were predominantly characterized by online delivery, active control conditions, young adult samples, and the use of negative stimuli (primarily facial images).

#### 3.3.2. Anxiety

**Figure 4:**
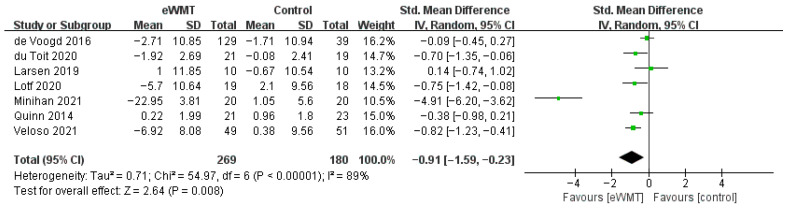
Forest plot of the effect of emotional working memory training on anxiety. Each square represents a study’s point estimate (size proportional to weight); the horizontal line shows the 95% confidence interval. Abbreviations: eWMT, emotional working memory training; IV, inverse variance; SD, standard deviation; CI, confidence interval [32,42,43,46,47,48,52].

Pooling seven studies revealed a significant overall reduction in anxiety favoring eWMT over controls (SMD = −0.87, 95% CI = −1.51 to −0.23, *p* = 0.008). However, heterogeneity was substantial (*I*^2^ = 89%). The CIs for the effect sizes of each study are presented in the forest plot (Figure 4).

Visual inspection identified the study by Minihan et al. [32] as an outlier (SMD = −4.91). A sensitivity analysis excluding this study yielded a smaller but significant pooled effect (SMD = −0.44, 95% CI = −0.23 to −0.17, *p* < 0.001) with markedly reduced heterogeneity (*I*^2^ = 51%), which represents a more reliable estimate of the intervention’s effect on anxiety.

Furthermore, subgroup analyses indicated that the results for the number of sessions, training time, and training frequency were not statistically significant.

#### 3.3.3. Depression

**Figure 5:**
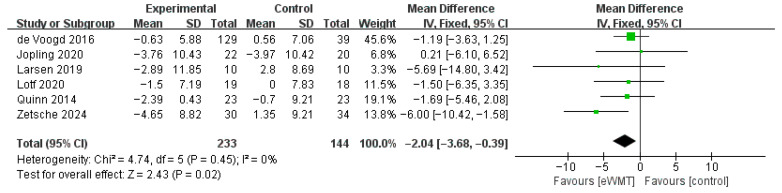
Forest plot of the effect of emotional working memory training on depression. Each square represents a study’s point estimate (size proportional to weight); the horizontal line shows the 95% confidence interval. Abbreviations: eWMT, emotional working memory training; IV, inverse variance; SD, standard deviation; CI, confidence interval [42,44,46,47,48,53].

Pooling six studies (n = 377) demonstrated a significant reduction in depressive symptoms, favoring eWMT over control conditions (Mean Difference = −2.04, 95% CI = −3.68 to −0.39, *p* = 0.02). Heterogeneity among the studies was negligible (*I*^2^ = 0%), indicating a highly consistent effect across the included trials. The CIs for the effect sizes of each study are presented in the forest plot (Figure 5).

#### 3.3.4. Rumination

**Figure 6:**
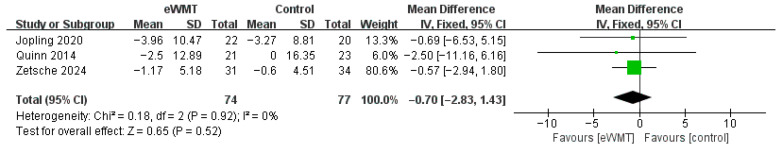
Forest plot of the effect of emotional working memory training on rumination. Each square represents a study’s point estimate (size proportional to weight); the horizontal line shows the 95% confidence interval. Abbreviations: eWMT, emotional working memory training; IV, inverse variance; SD, standard deviation; CI, confidence interval [44,48,53].

The meta-analysis of three studies (n = 151) found no significant effect of eWMT on rumination compared to control conditions (Mean Difference = −0.65, 95% CI = −2.61 to 1.31, *p* = 0.52). Heterogeneity was negligible (*I*^2^ = 0%), indicating a consistent pattern across the available evidence. (Figure 6).

#### 3.3.5. Reappraisal

**Figure 7:**
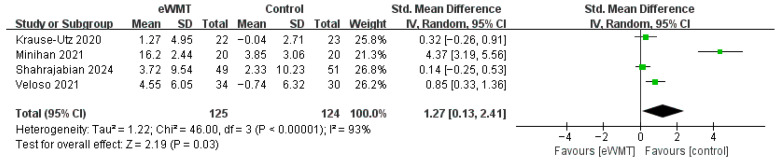
Forest plot of the effect of emotional working memory training on reappraisal. Each square represents a study’s point estimate (size proportional to weight); the horizontal line shows the 95% confidence interval. Abbreviations: eWMT, emotional working memory training; IV, inverse variance; SD, standard deviation; CI, confidence interval [32,45,51,52].

The meta-analysis of four studies (n = 249) initially indicated a significant but highly heterogeneous effect on reappraisal (SMD = 1.27, 95% CI = 0.13 to 2.41, *p* = 0.03; *I*^2^ = 93%). The study by Minihan et al. [32] was identified as an outlier (SMD = 4.37). Critically, a sensitivity analysis excluding this outlier provided a more robust estimate, revealing a significant, small-to-moderate effect (SMD = 0.38, 95% CI = 0.11 to 0.66, *p* = 0.006) with substantially reduced heterogeneity (*I*^2^ = 42%). This indicates a reliable, albeit smaller, benefit of eWMT on improving reappraisal capacity (Figure 7).

### 3.4. Publication Bias

Formal assessment of publication bias was conducted only for the working memory outcome, which included 12 studies, meeting the minimum threshold for reliable interpretation. Egger’s regression test indicated no statistically significant evidence of publication bias (z = 1.22, *p* = 0.22). Visual inspection of the funnel plot corroborated this finding, showing no substantial asymmetry. For all other outcomes—anxiety (7 studies), depression (6 studies), reappraisal (4 studies), and rumination (3 studies)—the number of included studies was below 10, precluding meaningful assessment of publication bias due to limited statistical power (Figure S1).

## 4. Discussion

The results of this meta-analysis, which included 16 randomized controlled trials with a total of 883 participants, indicated that eWMT is an effective intervention for enhancing working memory capacity (SMD = 0.47) and alleviating depressive symptoms (MD = −2.04), with significant and robust effects. Crucially, a significant effect on reappraisal was confirmed after sensitivity analysis removed an outlying study, yielding a smaller but robust and significant effect (SMD = 0.38). For anxiety, a significant overall effect was observed, but it was highly variable and reduced to a smaller yet significant level after correcting for an outlier. Finally, we found no significant effect of eWMT on rumination, though this conclusion is based on a very limited number of studies.

The enhancement in working memory capacity observed in this study represents a direct instance of a near transfer resulting from eWMT. This finding aligns with previous research indicating that computerized WMT can improve individuals’ working memory capacity [37]. Working memory capacity—the ability to retain and manipulate information—is strongly correlated with several measures of cognitive control [54,55]. Moreover, working memory plays a crucial role in cognitive development [56]. Thus, the increase in working memory capacity due to eWMT may facilitate cognitive processes such as cognitive flexibility, cognitive inhibition, and attention.

As mentioned in the Introduction, the capacity to regulate emotions plays a crucial role in influencing depression and anxiety levels, and maladaptive appraisal processes are central to this condition. Consequently, cognitive behavioral therapies for depression often emphasize reappraisal skills [57]. The present study found that individuals who underwent eWMT showed improvements in reappraisal scores, indicating the benefits of eWMT for emotion regulation. Specifically, the enhancement in cognitive reappraisal was attributed to the expansion of working memory capacity, which further boosts cognitive flexibility [58]. Additionally, although our meta-analysis of three studies did not find a statistically significant reduction in rumination following eWMT, the theoretical rationale for its potential impact remains noteworthy. Rumination, a maladaptive response pattern involving a repetitive and passive focus on the causes and consequences of distress, is a well-established cognitive process closely linked to depression [59]. Theoretically, eWMT aims to improve an individual’s ability to manage negative emotions by facilitating the clearance of irrelevant negative information from working memory [60]. It is plausible that this enhanced cognitive control could, in turn, contribute to a reduction in ruminative tendencies. The non-significant finding in the present analysis should therefore be interpreted with caution due to the very limited number of available studies, which severely constrains statistical power. Future research with larger samples is needed to adequately test the effect of eWMT on this clinically relevant cognitive process.

Our study observed reductions in both depression and anxiety symptoms following eWMT, supporting our hypothesis that eWMT can alleviate depression. Although the exact mechanism through which eWMT exerts this effect remains unclear, the results of the present study suggest that eWMT may enhance emotion regulation by improving working memory capacity, which in turn promotes cognitive flexibility and cognitive reappraisal ability while potentially reducing rumination. Enhanced emotion regulation, in turn, enables individuals to employ more effective strategies, such as cognitive reappraisal, and to exhibit less negative attentional bias toward adverse events.

The impact of eWMT on depression and anxiety through emotion regulation can be explained using a theoretical framework that categorizes emotion regulation into two types: model-based (strategic) and model-free (automatic) [61]. One hypothesis posits that eWMT enhances emotion regulation by increasing working memory capacity and improving executive functioning, which supports the model-based perspective. Specifically, cognitive reappraisal improvement is attributed to the expansion of working memory capacity, which further enhances cognitive flexibility. Moreover, eWMT helps manage negative emotions by removing irrelevant negative information from working memory, thereby reducing emotional load and, consequently, rumination, a strategy linked to depression [59]. Another hypothesis suggests that the training effect of eWMT relies on repeated exposure to emotional stimuli [49], facilitating automatic emotional adaptation and implicit emotion regulation (model-free). Our results, however, found no significant effect on rumination, indicating that this specific automatic pathway may not be a primary mechanism of eWMT. Thus, while effective implicit regulation could theoretically avert sustained negative affect, a hallmark of depression, the current evidence does not confirm that eWMT operates through this mechanism. Further research is required to clarify whether this model-free pathway is applicable to eWMT and to elucidate the precise mechanisms underlying its effects on depression and anxiety.

Our subgroup analyses provide a nuanced understanding of training intensity by separating the effects of training duration and dose concentration. The sensitivity analysis revealed that long-term training yields a reliable, moderate effect (SMD = 0.80, *I*^2^ = 0%), whereas a large training dose does not. This critical discrepancy suggests that the efficacy of eWMT is driven more effectively by distributed practice over time than by massed practice in a condensed format. The former likely allows for better consolidation of emotional regulation skills, while the latter may lead to cognitive fatigue or poor adherence. Therefore, protocol design should emphasize a sufficient number of distributed sessions rather than concentrating the training into high-dose, intensive bursts. Eventually, given the limited number of studies and the substantial heterogeneity in the data, extrapolation of our results should be approached with caution.

In summary, while our meta-analysis provides necessary preliminary evidence for the dual-path model, a more definitive test requires analytical approaches that can assess mediated pathways and their specificity (e.g., using individual participant data meta-analysis). Ultimately, confirming that eWMT’s efficacy is mediated by the postulated cognitive and emotional regulatory changes is essential for validating the theoretical model.

## 5. Limitations

This review has several limitations that warrant careful consideration. First, the limited number of studies included may not only introduce bias due to small sample sizes but also preclude robust subgroup analyses for testing specific hypotheses. A key example is the role of stimulus valence: although the theoretical framework emphasizes negative stimuli, the small number of studies using homogeneous valence types (e.g., exclusively negative, positive, or neutral) resulted in subgroups with fewer than three studies for some subgroups, which is methodologically insufficient for reliable comparative analysis. Second, publication bias remains a concern. The moderate to large effect sizes, combined with the small number of available studies for most outcomes, suggest that our pooled estimates might be inflated by unpublished null findings. Formal tests were underpowered to reliably detect bias, given the limited sample sizes. Therefore, the true efficacy of eWMT may be more modest than reported here. These findings should be considered provisional until supported by larger, preregistered studies that include the publication of null results. Third, despite our efforts to include a broad range of relevant variables, the efficacy of eWMT may not be fully accurate due to the diversity of psychological assessment tools used [36]. Fourth, specific choices made during our data extraction and synthesis process warrant consideration. To ensure comparability across studies, we prioritized outcome measures that were most commonly reported (e.g., selecting the Depression Anxiety and Stress Scale (DASS-21) for depressive symptoms when multiple scales were available). While this approach enhances consistency, it may inadvertently obscure effects that are unique to less frequently used, but potentially more sensitive, assessment tools. Finally, the characteristics of the study participants can influence research outcomes. Most participants in the included studies were female, which is noteworthy given that sex differences impact the generation, maintenance, and development of depression and anxiety as well as related brain structures and functions [57,62,63,64].

## 6. Conclusions

This meta-analysis aimed to address the efficacy of emotional working memory training (eWMT) for (1) enhancing working memory capacity, (2) improving emotion regulation processes (reappraisal and rumination), and (3) alleviating clinical symptoms (depression and anxiety), along with (4) the role of key moderating factors.

Our synthesis provides clear answers: eWMT effectively enhances working memory capacity and reduces depressive symptoms, with smaller but significant benefits for anxiety and reappraisal. However, it does not significantly reduce rumination—a finding that invites theoretical refinement. Critically, training intensity emerges as a key moderator: lower-intensity protocols yield more consistent benefits, while high-intensity results are highly variable, challenging a “more is better” assumption.

Thus, while supportive of the dual-path model, these results underscore the need for analytical approaches that can rigorously test the model’s specific mediated pathways. Future studies should therefore prioritize uncovering the active mechanisms and boundary conditions over dose–response questions. Demonstrating that efficacy is indeed mediated by the hypothesized cognitive and emotional changes remains the pivotal step for theoretical validation and intervention optimization.

## Figures and Tables

**Table 1:** Characteristics of included studies.

First Author and Publication Year	Country	Population	Sample Size (n)	Age, Years	Female (%)	Emotional Working Memory Training Paradigm	Training Duration	Training Environment	Control Condition	Working Memory Task	Emotional Task
Cantarella 2021 [20]	Italy	Healthy older adults	35	68.79	80.00	Criterion task: the Matrix task	8 times, 4 × 60 min each wk for 2 wk	Online	Active control	Forward and backward Corsi Span task	n.a.
de Voogd 2016 [42]	The Netherlands	High school adolescents in the Netherlands	168	14.35	60.12	Emotional N-back WMT	8 times, 2 × 15 min each wk for 4 wk	Online	Active control	Self ordered pointing task	Children’s Depression Inventory, Screen for Child Anxiety Related Emotional Disorders, Perseverative Thinking Questionnaire
du Toit 2020 [43]	Sydney	Adults with elevated social anxiety	40	22.95	87.50	Affective dual n-back task	6 times, 6 × 10 min each wk for 1 wk	Online	Active control	Digit span backward task	Peak anxiety rating scale
Jopling 2020 [44]	Canada	Participants with major depressive disorder	58	29.17	68.97	Affective version of the Sternberg task	6 times, 6 × 15–20 min each wk for 1 wk	Online	Active control	n.a.	Beck Depression Inventory, Second Edition Ruminative Responses Scale
Krause-Utz 2020 [45]	USA	Patients of Department of Psychosomatic Medicine and Psychotherapy	45	32.43	100	Affective dual n-back task	20 times, 7 × 20–30 min each wk for 3 wk	Online	Active control	n.a.	n.a.
Larsen 2019 [46]	USA	Veterans with elevated PTSD symptoms	20	52.79	25.00	Adaptive dual n-Back	15 times, 4 × 20 min each wk for 4 wk	Online	Active control	Operation span task	Depression Anxiety and Stress Scale
Lotf 2020 [47]	USA	Students from university of Wisconsin-Milwaukee and the surrounding Milwaukee area	37	23.79	75.68	Emotional N-back WMT	9 times, 5 × 20–25 min each wk for 2 wk	Online	Active control	Operation span task	Depression Anxiety and Stress Scale Penn State Worry Questionnaire
Minihan 2021 [32]	Wales	University students	40	20.55	50.00	Affective dual n-back task	20 times, 7 × 30–45 min each wk for 3 wk	Offline	Passive control	Backward version of the digit span task.	Test Anxiety Inventory
Pan 2018 [24]	China	Healthy university students from Beijing	33	20.57	48.48	Dual dimension n-back training based on smartphone app	5 times, 1 × 30 min each wk for 1 wk	Online	Active control	Backward version of the digit span task	n.a.
Pan 2020 [28]	China	University students in Beijing China with trait anxiety	65	21.94	73.85	Dual dimension n-back training application	21 times, 7 × 40 min each wk for 3 wk	Online	Active control	n.a.	Depression Anxiety and Stress Scale
Quinn 2014 [48]	USA	Undergraduate students	44	19.26	40.91	N-back task	1 time	offline	Active control	n.a.	n.a.
Schweizer 2011 [49]	UK	Undergraduate students	31	25	64.52	Dual n-back training task	20 times, 7 × 20–30 min each wk for 3 wk	Online	Active control	Forward digit span test	n.a.
Shahrajabian 2023 [50]	Iran	High school and university students	36	20.27	69.44	Affective dual n-back task	20 times, 3 × 30–45 min each wk for 7 wk	Online	Active control	Wechsler digit span test	n.a.
Shahrajabian 2024 [51]	Iran	Young adult with problem online sports bettors	64	34	30	Affective dual n-back task	20 times, 3 × 30–45 min each wk for 7 wk	Online	Active control	Wechsler digit span test	Cognitive emotion regulation questionnaire
Veloso 2021 [52]	Australia	Undergraduate students	100	27.41	51.00	Emotional dual n-Back WMT	20 times, 7 × 20 min each wk for 3 wk	Online	Passive control	Operation span task	Spielberger State-Trait Anxiety Inventory
Zetsche 2024 [53]	Germany	Participants with major depressive disorder	65	31	34	Adaptive emotional n-back task	10 times, 5 × 30–45 min each wk for 2 wk	Online	Passive control	Working memory selection task	Center for Epidemiological Studies Depression Scale, Ruminative Responses Scale—Short Form

Abbreviations, eWMT, emotional working memory training; n.a. not applicable; PTSD, post-traumatic stress disorder; WMT, working memory training.

**Table 2:** Subgroup analysis of working memory capacity.

Subgroup	No. Studies	Sample Sizes	SMD [95% CI]	Statistical Method	*p* Value	Heterogeneity (*I*^2^)
**Number of sessions**						
Long-term training	5 (4)	190 (150)	1.73 [0.51, 2.94] (0.80 [0.47, 1.13])	SMD (IV, random, 95%CI)	0.005 (<0.001)	92% (0%)
Short-term training	7	419	0.02 [−0.02, 0.07]	SMD (IV, fixed, 95%CI)	0.37	0%
**Training time**						
Large training dose	3 (2)	111 (71)	1.09 [−0.14, 2.32] (0.44 [−0.03, 0.92])	SMD (IV, random, 95%CI)	0.08 (0.07)	89% (0%)
Small training dose	9	503	0.29 [0.10, 0.47]	SMD (IV, fixed, 95%CI)	0.03	39%
**Training frequency**						
High training frequency	7 (6)	285 (245)	0.35 [0.11, 0.58] (0.32 [0.08, 0.57])	SMD (IV, fixed, 95%CI)	0.004 (0.01)	48% (54%)
Low training frequency	5	303	0.29 [0.04, 0.54]	SMD (IV, fixed, 95%CI)	0.02	0%
**subject type**						
Clinical population	4	183	0.37 [−0.19, 0.93]	SMD (IV, random, 95%CI)	0.20	70%
General population	8 (7)	425 (385)	0.53 [0.09, 0.97] (0.28 [0.06,0.49])	SMD (IV, random, 95%CI)	0.02 (0.01)	75% (0%)

Abbreviations: IV, inverse variance; SMD, standardized mean difference; CI, confidence interval. Values in parentheses represent sensitivity analysis results after excluding Minihan et al. [32].

## Data Availability

No new data were created or analyzed in this study. Data sharing is not applicable to this article.

## Supplementary Materials

The following supporting information can be downloaded at https://www.mdpi.com/article/10.3390/brainsci16010030/s1, Figure S1: Funnel Plots for Meta-Analyses; Table S1: The specific search strings; Table S2: PRISMA checklist. Reference [65] is cited in the supplementary materials.
