# Supplementary material for: Does Emotional Working Memory Training Ameliorate Anxiety and Depression? A Meta-Analytic Review
Source: Brain Sci. 2025 Dec 25;16(1):30. doi: 10.3390/brainsci16010030 (PMC12839229; doi:10.3390/brainsci16010030)
Supplement: Supplementary file 1 [file brainsci-16-00030-s001.zip › brainsci-4058290-supplementary/Figure S1 Funnel Plots for Meta-Analyses.pdf]

**Figure S1: Funnel Plots for Meta-Analyses**

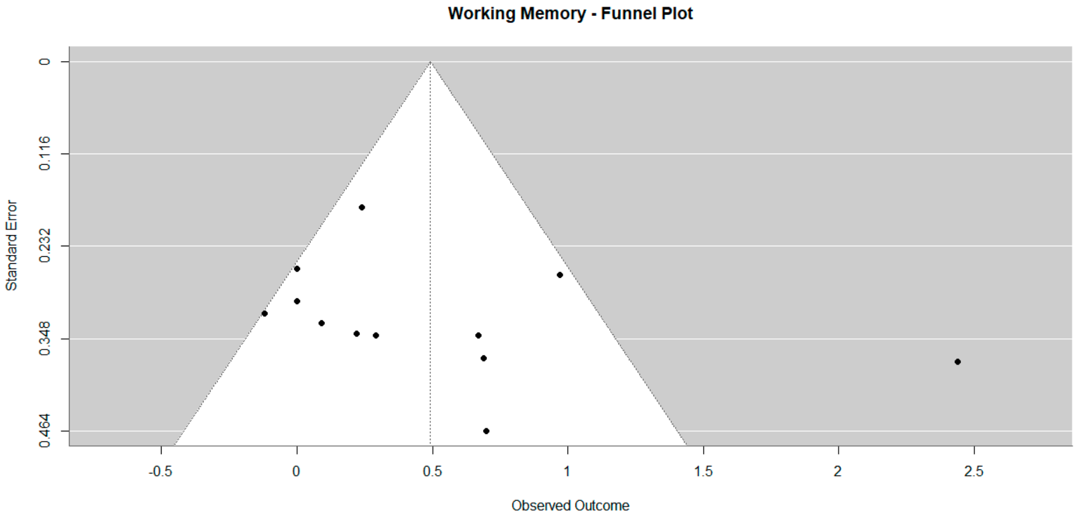


Figure S1. Funnel plots for eWMT on working memory capacity
