# Supplementary material for: Does Emotional Working Memory Training Ameliorate Anxiety and Depression? A Meta-Analytic Review
Source: Brain Sci. 2025 Dec 25;16(1):30. doi: 10.3390/brainsci16010030 (PMC12839229; doi:10.3390/brainsci16010030)
Supplement: Supplementary file 1 [file brainsci-16-00030-s001.zip › brainsci-4058290-supplementary/Table S1 The specific search strings.pdf]

Table S1: The specific search strings

Database	search strings
**Web of science**	((AB=(emotional OR affective)) OR (TI=(emotional OR affective))) AND ((AB=(working memory training OR n-back training OR memory updating training OR switching training OR shifting training OR dualtasking training)) OR (TI=(working memory training OR n-back training OR memory updating training OR switching training OR shifting training OR dualtasking training)))
**Pubmed**	((("Emotions"[Mesh]) OR (affective [Title/Abstract])) AND ((working memory training[Title/Abstract]) OR (n-back training[Title/Abstract]) OR (memory updating training[Title/Abstract]) OR (switching training[Title/Abstract]) OR (shifting training[Title/Abstract]) OR (dualtasking training[Title/Abstract])))
**PsycINFO**	(TI (affective OR emotional) OR AB (affective OR emotional))AND (TI (working memory training OR n-back training OR memory updating training OR switching training OR shifting training OR dual tasking training) OR AB (working memory training OR n-back training OR memory updating training OR switching training OR shifting training OR dual tasking training))
**Science Direct**	Title, abstract or author-specified keywords:(affective OR emotional) AND "working memory" AND training
